# Interviews with Indigenous Māori with type 1 diabetes using open-source automated insulin delivery in the CREATE randomised trial

**DOI:** 10.1007/s40200-023-01215-3

**Published:** 2023-03-23

**Authors:** Mercedes Burnside, Tracy Haitana, Hamish Crocket, Dana Lewis, Renee Meier, Olivia Sanders, Craig Jefferies, Ann Faherty, Ryan Paul, Claire Lever, Sarah Price, Carla Frewen, Shirley Jones, Tim Gunn, Benjamin J. Wheeler, Suzanne Pitama, Martin de Bock, Cameron Lacey

**Affiliations:** 1 Department of Paediatrics, University of Otago, Christchurch, New Zealand; 2 Department of Māori Indigenous Health Innovation (MIHI), University of Otago, Christchurch, New Zealand; 3 Te Huataki Waiora School of Health, University of Waikato, Hamilton, New Zealand; 4 OpenAPS, Seattle, WA USA; 5 Department of Paediatric Endocrinology, Starship Children’s Health, Te Whatu Ora Te Toka Tumai, Auckland, New Zealand; 6 Liggins Institute and Department of Paediatrics, University of Auckland, Auckland, New Zealand; 7 Waikato Regional Diabetes Service, Te Whatu Ora Health New Zealand Waikato, Hamilton, New Zealand; 8 Department of Women’s and Children’s Health, Dunedin School of Medicine, University of Otago, Dunedin, New Zealand; 9 Nightscout New Zealand, Hamilton, New Zealand

**Keywords:** Diabetes Mellitus Type 1, Health Equity, Qualitative Research, Automated Insulin Delivery, Open-Source, Indigenous Māori

## Abstract

**Purpose:**

Open-source automated insulin delivery (AID) is used by thousands of people with type 1 diabetes (T1D), but has unknown generalisability to marginalised ethnic groups. This study explored experiences of Indigenous Māori participants in the CREATE trial with use of an open-source AID system to identify enablers/barriers to health equity.

**Methods:**

The CREATE randomised trial compared open-source AID (OpenAPS algorithm on an Android phone with a Bluetooth-connected pump) to sensor-augmented pump therapy. Kaupapa Māori Research methodology was used in this sub-study. Ten semi-structured interviews with Māori participants (5 children, 5 adults) and whānau (extended family) were completed. Interviews were recorded and transcribed, and data were analysed thematically. NVivo was used for descriptive and pattern coding.

**Results:**

Enablers/barriers to equity aligned with four themes: access (to diabetes technologies), training/support, operation (of open-source AID), and outcomes. Participants described a sense of empowerment, and improved quality of life, wellbeing, and glycaemia. Parents felt reassured by the system’s ability to control glucose, and children were granted greater independence. Participants were able to use the open-source AID system with ease to suit whānau needs, and technical problems were manageable with healthcare professional support. All participants identified structures in the health system precluding equitable utilisation of diabetes technologies for Māori.

**Conclusion:**

Māori experienced open-source AID positively, and aspired to use this therapy; however, structural and socio-economic barriers to equity were identified. This research proposes strength-based solutions which should be considered in the redesign of diabetes services to improve health outcomes for Māori with T1D.

**Trial Registration:** The CREATE trial, encompassing this qualitative sub-study, was registered with the Australian New Zealand Clinical Trials Registry (ACTRN12620000034932p) on the 20^th^ January 2020.

**Supplementary Information:**

The online version contains supplementary material available at 10.1007/s40200-023-01215-3.

## Introduction

Health inequities are “differences which are unnecessary and avoidable, but in addition are considered unfair and unjust” [1]. Health inequities based on ethnicity are well reported worldwide [2], and arise from societal structures which restrict access to the social determinants of health [3]. In Aotearoa/New Zealand (referred as New Zealand herein), health inequities between Māori (Indigenous Peoples) and New Zealand Europeans (NZE) are the most compelling [4, 5]; life expectancy for NZE is 8 to 9 years longer, NZE are burdened with a lower prevalence of certain diseases [1, 6], and NZE have greater access to quality healthcare despite lower health needs [7]. The domineering research narrative for Māori health disadvantage has purported that Māori are the loci of negative health outcomes due to inferior genetics, intellect, behaviour, or aptitude [1, 8]. This type of colonial framing, or ‘deficit theory’, shrouds NZE privilege and masks the accrued preferential benefit from the design and continued control of Western health paradigms. However, Māori have resisted this cultural-deficit narrative and continue to advance research in Māori health [9].

Type 1 diabetes (T1D) is an exemplar of a health condition whereby marginalised ethnic groups are over-represented in poorer health outcomes worldwide [10]. While NZE account for 75.8% of people with T1D (followed by Māori (10.1%), Asian (6.5%), Pacific (4.2%), and people of other ethnicities (1.4%) [11]), evidence supports a growing burden in marginalised ethnic groups [12]. Further, Māori with T1D are at greater risk of developing long-term complications with non-optimal glycaemia compared to NZE irrespective of socioeconomic status [13]. Access to publicly funded insulin pump therapy also favours NZE [11, 14], and NZE are less likely to have insulin pump therapy withdrawn [15] due to access criteria. Insulin pumps are publicly funded in New Zealand, but only those with a glycated haemoglobin (HbA1c) between 65 and 90 mmol/mol (8.1%—10.4%) are eligible [16]. These criteria systematically disadvantage Māori who do not qualify for the technology based on HbA1c. Continuous glucose monitoring (CGM), another important diabetes technology proven to improve glycaemia [17], is not funded in New Zealand, either publicly or through health insurance. Burnside et al. [18] found CGM use is highest amongst NZE, and universal access to CGM is one way to reduce inequities in glycaemic outcomes between ethnic groups.

Automated insulin delivery (AID) systems, comprising a control algorithm, insulin pump, and CGM, consistently improve glycaemia and reduce management burden for people with T1D [19]. Despite several commercial systems now being available, access varies markedly depending on regional regulatory approval and funding, and insurance and reimbursement policies. A community movement has emerged in T1D which aims to reduce inequities in access to AID through user-centred innovation. A do-it-yourself (DIY) AID system was developed by people with diabetes and shared freely as an open-source system before commercial systems became available [20]. The founders freely shared the algorithm, named OpenAPS, as an open-source system, and it continued to evolve with additional community input. Open-source AID has been repeatedly studied in real-world and retrospective or prospective settings [21]. However, previous AID literature is limited by lack of reported patient demographic characteristics including ethnicity. Huyett et al. found that only six of 99 commercial AID studies reported on ethnicity [22] making it difficult to ascertain the range of people using an AID system and hence its wider applicability.

Before AID can be considered a way to address health inequity, it is necessary to investigate how marginalised ethnic groups experience this therapy. Therefore, the aim of this qualitative study was to explore the experiences of Indigenous Māori participants with use of an open-source AID system to identify cultural, structural, socioeconomic, and clinical enablers/barriers to health equity. Further, this study proposes solutions to identified barriers that are informed by the experiences of Māori and their whānau (extended family).

## Methods

### Research approach and paradigm

Kaupapa Māori Research (KMR) methodologies informed this qualitative work [23] (Supplemental Table 1). The KMR framework was developed “by Māori, for Māori” [8], and hence is distinctive to New Zealand. This research paradigm is informed by a Māori world view where being Māori is normal, and there is Māori control over the design, data collection and analysis, and interpretation of findings. Pervasive health inequities for Māori with T1D provide a strong rationale for privileging the voices of Māori through the application of KMR methods.

### Context

This qualitative study was conducted as part of a wider research project called the CREATE (**C**ommunity de**R**iv**E**d **A**utoma**TE**d insulin delivery) randomised controlled trial. The CREATE trial was an open-labelled, randomised (1:1), parallel-group, 24-week superiority trial evaluating safety and efficacy of an open-source AID system (OpenAPS algorithm [24] in a modified version of the AndroidAPS application on an Android phone, pre-production DANA-i insulin pump, and Dexcom G6 CGM) in 97 children and adults (7 – 70 years) with T1D. A 24-week continuation phase followed to assess long-term outcomes. The CREATE trial was conducted across four New Zealand sites, and staff provided 24/7 clinical and technical support to participants. A detailed description of the trial protocol [25] and results from the trial have been published elsewhere [26].

### Sample

The CREATE trial prioritised recruitment of Māori participants to ensure population representation [11]. Fourteen of the 97 (14.4%) participants randomised in the CREATE trial self-identified as Māori. All 14 Māori participants were invited to be interviewed.

### Ethics

The CREATE trial, encompassing this qualitative study, is registered with the Australian New Zealand Clinical Trials Registry (ACTRN12620000034932p) and was approved by the Southern Health and Disability Ethics Committee (20/STH/1). Written informed consent (or assent from minors aged < 16) was obtained from all participants and parents/guardians of minors prior to participation. This research was conducted in accordance with Health Research Council of New Zealand guidelines for Māori research [27] and integrates KMR methodologies to ensure the research is responsive to Māori.

### Procedure

Semi-structured interviews utilised an interview schedule with key discussion points rather than specific questions. This approach suited the exploratory nature of the research aim and aligned with KMR methodology [23] by positioning participants as experts and exploring topics of salience to them. The interview schedule was informed by; a literature review (conducted by MB for doctoral thesis), input from clinical and Indigenous health colleagues, as well as input from a qualitative researcher with expertise on open-source AID. Topics were designed to seek out cultural, structural, socioeconomic, and clinical enablers and barriers to equity for Māori participants with T1D (Table 1).

**Table 1:** Interview schedule (key topics)

Pre-trial experiences	Diagnosis Ongoing management Accessing diabetes technology and research
Experiences using the open-source AID system	Navigating the AID system Ease of use of the various functions able to be accessed by participants Challenges/rewards Influences impacting on use/or understanding of the system Technical aspects- managing hardware components, troubleshooting
Clinical impact	How it influenced diabetes self-management Day to day glucose levels Glycaemic outcomes
Impact on daily life/whānau life	Quality of life Wellbeing for person with T1D and whānau (physical, emotional, mental, spiritual, relationships with others)
Views on training/educational resources/ongoing support	AID training and ongoing learning after AID initiation Different forms of support – healthcare professional, online peer support, training materials (written guides, ‘how to’ videos)

### Data collection and processing

Interviews took place within six weeks of participants completing 24 weeks of open-source AID use, with the exception of one child participant interviewed after 12 weeks of use due to withdrawal from the CREATE trial. This allowed time for the technology to become entrenched in daily whānau living. All ten interviews were facilitated by MB, a Māori researcher and clinical expert on the CREATE trial study team, between June 2021 and April 2022. Participants were interviewed with their whānau at a time and place they chose. All interviews were intended to be face to face; however, seven interviews were conducted via Zoom due to COVID-19 restrictions in New Zealand at the time. Interviews, ranging from 30 – 50 min, were audio-recorded and transcribed verbatim. Transcripts were anonymised assigning a number to each participant (adult 1–5, child 1–5, and parent 1–5).

### Data analysis

Data were analysed by MB, TH, and CL using an inductive thematic approach; the most frequently chosen analytical method among qualitative KMR literature at the time [28]. MB transcribed the first two interviews and the remaining eight transcripts were transcribed by an independent transcriber. Participants were given the opportunity to review transcripts and make changes prior to analysis. MB read all transcripts repeatedly to become familiar with the data. Data were coded using NVivo (QRS International Pty Ltd, 2014). Descriptive codes were developed inductively; MB undertook initial open coding, and this was followed by a process of pattern coding [29]. The resultant coding framework was reviewed by TH and CL, Māori health researchers, and MB completed a second cycle of pattern coding to ensure there was a distinct Kaupapa Māori orientation that considered the role of systemic factors in participants’ experiences of T1D care (Supplemental Fig. 1). Following an iterative process of discussion and review of coding by MB, TH, and CL, consensus on themes and subthemes were reached. Measures to enhance trustworthiness and credibility of the analysis included member checking and audit trail. No new insights emerged in the coding of the final interview, suggestive of data saturation.

### Data display

A summary of descriptive subthemes evident within the themes of Access, Training/Support, Operation, and Outcomes is provided. For each descriptive subtheme, a precis of participants’ comments, including critiques about the health system, is reported and supporting quotations are presented in Table 2. Table 3 presents barriers to equity synthesised from participants’ critique, along with some proposed solutions taken directly from transcripts or inferred from identified barriers.

**Table 2:** Participant quotations

Theme and Subthemes	Participant quotation
**Access**	
Procurement (|)	**Parent #4:** *“At that time pumps weren’t that popular, but the District Health Board (Health Service) had them. I had this big speech ready to ask for one because you had to prove [to the healthcare professional] that you could handle it before you got one.”*
Funding (|)	**Parent #1:** *“Dexcom was just too expensive for us all the time, and we would get really angry if she knocked it out, and it’s not fair that she gets in trouble for accidents.”*
Complexity (|)	**Parent #2:** *“The idea of learning all the stuff that comes with a pump was just too much at that point, and then once we got it we were really glad we did because it was lifechanging.”*
**Training/Support**	
Healthcare professional (*)	**Parent #4:** *“I’m a kinaesthetic learner, so I found the Healthcare professional ringing and talking me through it was a lot easier than the video or written guides.”*
Educational material (*)	**Parent #1:** *“It was good to know that they [educational materials] were there. II did read them before we went in and having prior knowledge was helpful.”*
Online peer support (|)	**Adult #4:** *“With Tribe, I’m relying on people I don’t know, I don’t know their background, and I don’t know their training. So, I didn’t have the time or energy to engage with the Tribe community.”*
**Operation**	
Trust (*|)	**Adult #3:** *“I did have a little bit of anxiety for the first 24 to 48 h. I was obsessed with looking at my phone and worried that it wasn’t going to do the right thing. Then I realised that it was actually doing a better job than what I do.”*
Adapting (*|)	**Adult #2:** *“It might take a bit of learning to get used to the system, but once you’ve learned, day to day it’s just a lot easier.”*
Technology (*|)	**Adult #5:** *“It’s [loss of Bluetooth connection between devices] a lot for me, it would go at least once a day if not more.”*
**Outcomes**	
Glycaemia (*)	**Adult #1:** *“It was never an achievable goal to have good blood sugar. I tried, but was never good enough and I was told “this is a bad number.” The biggest thing for me was achieving the best HbA1c I have ever had.”*
Wellbeing (*)	**Parent #5:** *“The huge benefit is being able to get up every morning and her glucose is five [mmol/L], and then looking back and seeing that she’s been doing this all night. Just a huge relief.”*
Empowerment (*)	**Adult #4:** *“Because it was immediate feedback I could see if it was working and it felt good.”*

(*) denotes a potential enabler

(|) denotes a potential barrier

Language used is framed to reflect the mana (prestige) of the Māori participants/whānau

**Table 3:** Barriers to equity and solutions by theme/subtheme

Access
a. Equitable procurement of insulin pumps and CGM devices for Māori will be achieved by: • Forsaking public funding access criteria to insulin pumps to ensure insulin pump therapy is an option available to all Māori • Including diabetes technology advocacy as a service specification for diabetes centres nationwide • Providing diabetes clinical teams nationally with appropriate training on diabetes technologies and resourcing to offer such therapies to Māori following a diagnosis of T1D • Including diabetes clinical teams in targeted interventions to improve their provision of effective health literacy information about diabetes technologies to all Māori/whānau to improve access
b. Costs of CGM for Māori/whānau will be addressed by: • Making publicly-funded CGM universal to Māori following a diagnosis of T1D • Prioritising universal funding of rt-CGM, over is-CGM, to address inequities in diabetes health outcomes, and allow Māori to access and benefit from AID systems
c. Clinical teams will improve perceived acceptability of diabetes technologies to promote uptake by: • Exploring Māori/whānau concerns related to the use of diabetes technologies, and partnering with Māori/whānau to address such concerns • Providing training and support tailored to the needs of Māori/whānau. This includes flexible appointment scheduling, home based training/support with whānau, access to a range of training materials to suit different learning styles, and access to 24/7 clinical and technical support using communication mediums suited to the Māori person/whānau
**Operation**
a. Clinical teams will help Māori/whānau foster trust in AID systems through: • The provision of training on how the control algorithm operates and makes decisions. Māori/whānau will be informed of certain algorithm operations known to raise doubt (such as the inclusion of rescue carbohydrate in future bolus recommendations). For Māori/whānau considering an open-source AID system, clinical teams will provide reassurance that new additions to the portfolio of pumps that can be used in open-source AID are not susceptible to ‘hacking,’ and that to date there are no reported cases of intentional harm or personal data breaches • Supporting Māori/whānau to optimise core user-specific settings so they are able to benefit the most from AID. This may require an additional level of clinical support during the first weeks of AID use
b. Clinical teams will support Māori/whānau affected by T1D to adapt to the AID treatment paradigm by: • Identifying specific functions of standard pump therapy (including the context) the person/whānau found useful, and imparting knowledge on how to replicate outcome(s) using the AID system • Recognising that Māori/whānau may require greater clinical (to optimising settings) and technical (to troubleshoot technical issues) assistance while they learn to navigate the AID system. Therefore, additional contacts with clinical staff, that are motivated by Māori/whānau needs, are available • Publishing and sharing the positive narratives of Māori/whānau who have previously learned to use AID
c. Māori/whānau affected by T1D will be supported by clinical teams to manage the technical aspects of AID through: • Quality in-person training on AID that is responsive/customised to the needs of Māori/whānau. This includes flexible scheduling of the training session(s) (date, time, location, duration e.g. over one or two days), and an optional probationary period where Māori/whānau have the opportunity to use the new hardware components before initiating automation of insulin delivery • Ensuring teams are resourced to work with the Māori/whānau ongoing, providing wraparound support for the integration of technology into other competing priorities and restrictions. This may involve outreach/in-home supports • Recognition that technical issues frequently occur outside of working hours, and 24/7 technical support from staff will be readily available to Māori/whānau by phone • The availability and provision of a range of training materials (video demonstrations, printed and digital written guides) – these are not intended to replace support by clinical staff

## Results

**Fig. 1:**
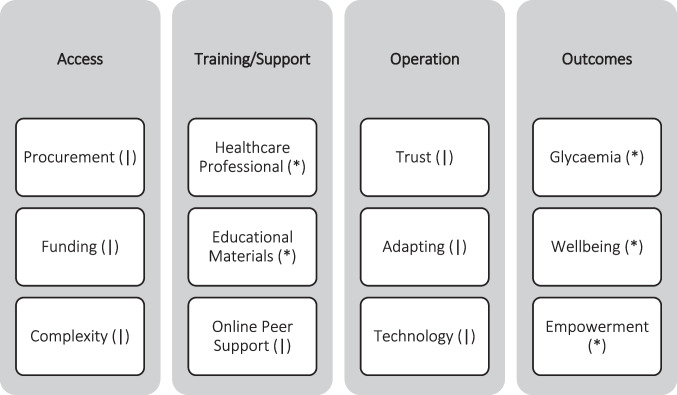
Themes and subthemes informed by participants’ comments about diabetes technologies, represented as potential enablers (*) or barriers (|)

The study sample comprised 5 adult participants (23 – 47 years), 5 child participants (10 – 16 years), and 5 whānau members from 4 child participants. Four of the 14 Māori participants in the CREATE trial who did not respond to invitations to be interviewed for reasons unknown to the researchers. Participant socio-demographic characteristics are presented in Table 4. Four themes were identified describing enablers and barriers to equitable utilisation of diabetes technologies for Māori participants. Three subthemes within each theme emerged from the analysis (Fig. 1).

**Table 4:** Socio-demographic characteristics of *n* = 10 Māori children and adults with T1D within the CREATE trial

Age group	***N* = 10**
- Child	5 (50%)
- Adult	5 (50%)
Median age, (IQR) – yr	19.5 (12, 36)
Gender – no. (%)	
- Female	7 (70%)
Highest qualification (parent of children) – no. (%)	
- Unknown - None - School (1–4) - Dip. or Cert. (5–6) - Graduate (7) - Postgraduate (8–10)	2 (20%) 2 (20%) 1 (10%) 1 (10%) 3 (30%) 1 (10%)
Income (household) – no. (%)^a^	
- Unknown - < $70,000 - $70,001—$100,000 - $100,001—$150,000 - $150,001 or more	1 (10%) 5 (50%) 1 (10%) 2 (20%) 1 (10%)
New Zealand Deprivation Index (quintile) – no. (%)^b^	
- 1 - 2 - 3 - 4 - 5	2 (20%) 2 (20%) 2 (20%) 2 (20%) 2 (20%)
Study Site – no. (%)^c^	
- Christchurch - Dunedin - Hamilton - Auckland	2 (20%) 3 (30%) 3 (30%) 2 (20%)
Initial Randomisation	
- Open-source AID - Sensor augmented pump therapy	4 (40%) 6 (60%)
Time in range at baseline (%), mean (SD)^d^	53% (17)
Time in range at study end (%), mean (SD)^d^	60% (11)

^*^Socio-demographic characteristics of whānau/parents not captured

^a^Annual household income in New Zealand dollars

^b^The New Zealand deprivation index is an area-based measure of socioeconomic deprivation in New Zealand where quintile 5 represents the 20% most deprived areas in New Zealand

^C^Geographic location of participants residence. Christchurch and Dunedin are in the South Island and Hamilton and Auckland, the North Island of New Zealand

^d^Percentage time with sensor glucose level in target range (3.9-10 mmol/L [70-180 mg/dL])

### Access

The Access theme identified participants’ tribulations accessing diabetes technologies outside of funded trials, including (publicly funded) insulin pumps and (non-funded) CGM devices; both essential hardware components of AID systems. Three subthemes were extrapolated from the Access theme including participant critiques about: *Procurement* of technology; lack of technology *Funding*; and perceived *Complexity*.

#### Procurement

Procurement was defined by participants’ critique of ascertaining and sourcing diabetes technologies outside of the study. Participants commonly learned about diabetes technologies online, and due to technologies only becoming available, funded or unfunded, years after other OECD countries, many reported challenges importing CGM from overseas. One parent noted the present-day challenge of sourcing hardware to build an open-source AID system. Participants did not have difficulty qualifying for a publicly funded insulin pump, but most were mindful of having to meet criteria to retain them, and two participants reported significant worry arising from the threat of having to surrender their pumps. Participants provided examples where healthcare professionals functioned as gatekeepers to technology by not advertising available technologies, or by having to prove their ability and lobby to a healthcare professional to gain access.

#### Funding

Funding was reported within participants’ critique of the lack of funded CGM in New Zealand, and the high cost to self-fund it outside of the trial. Young adults often did not explore CGM as a therapy due to cost prohibition. Participants from the parent cohort described extreme measures to fund CGM for their children including various fundraising endeavours, Givealittle (online platform for crowdfunding), and sponsorship. They also described purchasing CGM devices whenever their financial situation would allow. Prior to the trial it was common for whānau to ration the use of CGM, for example prioritising use to gauge the effect of changes to insulin delivery settings. Further, participants from the parent cohort reported cost-related stress, then guilt for scolding their child if a CGM sensor was accidentally displaced.

#### Complexity

Complexity was explained by participants’ critique of learning how to use insulin pumps and CGM in their technology experiences previous to the trial. In some cases, participants delayed uptake of insulin pump therapy by years due to perceived complexity and stress associated with learning the technology. Participants noted the need to learn to count the carbohydrate content of foods as a consideration. Two adult participants were reluctant to adopt a pump due to concerns with being attached to a device constantly. Some participants had trialled then abandoned an open-source device that converts intermittently scanned CGM (isCGM) to real-time CGM (rtCGM) due to a lack of support to troubleshoot technical issues. Time, or lack of, was also raised as a barrier to learning diabetes technologies if perceived to be complex.

Despite the barriers to access reported above, all participants described overwhelmingly positive experiences with insulin pumps and CGM, going as far to pronounce the technologies as life changing. Key benefits pertained to greater convenience and reduced user burden, tolerability (especially for children), and freedom. Access to a healthcare professional who advocated diabetes technologies strongly influenced technology utilisation by participants prior to the trial.

- Child #3: “I got a pump when I was diagnosed because she [healthcare professional] got me onto one straight away.”

### Training/Support

The Training/Support theme captured participants’ views on open-source AID training and support within the CREATE trial, applicable to the training/support likely to be necessary in the real-world for AID. From the analytical process it was apparent that even within this small cohort, participant needs differed – some found a single training day ample and gained little from the run-in period (4-week period using the study devices without AID). Comparatively, some participants left the initial training session feeling overwhelmed and said the run-in period alleviated some of the stress of new diabetes devices.

Three subthemes were identified including participant sentiments about: the importance of training/support by a *Healthcare Professional*; the appeal of having access to *Educational Materials*; and the limited usefulness within the trial of *Online Peer Support*.

#### Healthcare Professional

Healthcare Professional was captured through participants’ compelling sentiments that access to a healthcare professional for initial training and ongoing technical/clinical support is a requisite for successful adoption of AID. Participants mainly sought the support of their healthcare professional while learning to navigate the system and troubleshoot technical issues. Contacts via text message, email, or phone call were common, and in-person support was rarely required. Participants preferred this form of support because it was convenient and available 24/7, they trusted and understood the advice, and valued having another person to share their concerns and support decision making.

#### Educational Materials

All participants in the CREATE trial were provided with written guides on AID, and ‘how to’ video demonstrations (all in English). The subtheme of Educational Materials involved participants’ reflections about the usefulness of these materials. Opinions on usefulness varied, but most commented that it was reassuring knowing they were readily available to them, and some found reviewing the materials prior to the initial training helpful. Participants liked that they were provided with printed and digital copies of written guides.

#### Online Peer Support

Participants in the CREATE trial were invited to join a closed online community (Tribe Technologies Inc.) for peer support to simulate the community support that is used by real-world open-source AID users. This subtheme involved participant critiques of the online community within the trial. Participants did not find the online community useful in the context of this trial for reasons including: the community lacked momentum and information; some technical issues warranted urgent intervention; many did not engage in social media; and they instead preferred approaching their healthcare professional who was familiar, trusted, approachable, and able to give immediate advice.

### Operation

The Operation theme documented participants’ experiences operating the open-source AID system in daily whānau life. Three subthemes were identified from the Operation theme including participant critiques about: lack of *Trust* in the system; *Adapting* to a new treatment paradigm; and managing *Technology*.

#### Trust

Trust included participants’ critique of the open-source system automating insulin delivery. Adult and parent participants described feeling ambivalent about relinquishing control, reporting an initial probationary period (days) when they scrutinised the application’s graphs for reassurance that its decisions were safe and correct. Some admitted to overriding the system if they had any doubts about its functioning, and noted, in hindsight, that this was counterproductive. Participants also described developing trust in the system as a result of seeing the algorithm making logical decisions and responding to glucose excursions quicker than they could. Some instances of mistrust were seeded by a lack of understanding of how the algorithm made treatment decisions, for example, the inclusion of rescue carbohydrates (announced to the system) in future insulin bolus recommendations. One adult participant expressed concern that the open-source system could be vulnerable to hacking. This concern was not shared by other participants, including those with prior experience with open-source innovations.

#### Adapting

Adapting comprised participants’ critique of adapting to a new treatment paradigm. Participants reported using their ‘usual diabetes care’ for several years and described challenges (taking time, effort, trial and error) adapting to new ways of thinking and performing diabetes self-care. Many described a struggle to replicate aspects of their usual care and one participant found her low carbohydrate ketogenic diet led to ketosis with AID. Some reported it took time to tweak core insulin settings, and some even described manipulating the system for preferred insulin delivery, for example, announcing additional carbohydrates to liberate the system (this was reported by two adults with highest total daily dose of insulin). One adolescent participant found the change of paradigm overwhelming, preferring to administer insulin through her insulin pump instead of the AID application, and withdrew after three months of open-source AID use. Despite the challenges voiced, participants had an overwhelming consensus was that it is well worth the initial effort to learn to use the open-source AID system.

#### Technology

Technology took account of participants’ critique of maintaining hardware components of the AID system, and troubleshooting technical issues. Participants perceived building an open-source AID system from scratch to be technically challenging, noting the provision of a pre-built system as a benefit of the trial. Increased technical troubleshooting (of hardware and connectivity issues rather than AID troubleshooting) was raised ubiquitously. Whilst technical issues were described as stressful, most participants regarded the degree of troubleshooting to be manageable with healthcare professional support. Participants described mild burden associated with maintaining the hardware, for example, charging the Smartphone daily, and child participants found it especially difficult to carry the Smartphone constantly.

Importantly, operational difficulties conveyed by participants mainly related to technical aspects, and use of the application interface on the Smartphone itself was described as easy to use, even by the youngest participant aged 10. Participants enjoyed the information presented on application home-screen (glucose level and future glucose prediction lines), and accessing a wide array of settings and features which allowed for pin point management. Unanticipated benefits included the ease of adjusting insulin delivery settings and discrete insulin bolus administration through the Smartphone.

- Adult #2: “I found it really intuitive, I really liked the display.”

### Outcomes

The Outcomes theme identified narrated benefits of AID. Three subthemes were ascertained from the Outcomes theme including participant sentiments about: improved *Glycaemia*; improved *Wellbeing*; and a sense of *Empowerment*.

#### Glycaemia

Glycaemia acknowledged participants’ sentiments about improved blood glucose outcomes. Many participants reported meaningful reductions in HbA1c (especially participants with the highest HbA1c prior to AID). Others described being able to maintain a low HbA1c with less effort. Participants found AID reduced hypo/hyperglycaemia and glucose fluctuations by providing levels of responsiveness beyond their own capabilities, especially when they were distracted by other responsibilities during the day. Every participant mentioned the positive effect of AID on nocturnal glucose levels, which would invariably also translate to a better day. AID was also reported to aid optimal glucose levels during exercise.

#### Wellbeing

Wellbeing contained participants’ sentiments about the holistic benefits of open-source AID use. Participants articulated that the system reduced the burden of diabetes self-management allowing them to experience life more normally. Child participants liked that they no longer needed to finger prick, adolescents could get away with missed meal bolus administration, and adult participants liked that the system did the thinking for them. AID improved mood and cognition, and reduced worry, including the daily fear of dying from T1D for one participant. Participants also reported improved sleep and energy. For participants who were parents, CGM alarms and the ability to follow glucose levels remotely ameliorated the need to constantly monitor their child, and children were granted independence. AID enabled prompt management of glucose excursions, and consequently parents experienced less guilt.

#### Empowerment

Empowerment encompassed participants’ sentiments about playing a greater role in the management of their diabetes. The open-source AID system endowed participants with real-time glucose data and additional diabetes data which empowered them to think critically about their diabetes self-care and collaborate more with this system than previous therapies. Participants relished self-governance; adjusting user-specific settings and observing the outcomes.

## Discussion

This study employed KMR methodology to cast a health equity lens on diabetes technology utilisation by Māori with T1D in New Zealand. Participants identified enablers and barriers to equity which aligned with the four key themes of access, training/support, operation, and outcomes. Participants described holistic benefits of open-source AID, and their aspirations to continue using the AID system beyond the trial. Although participants experienced an open-source AID system in this study, they may also experience commercially available systems positively since these alternatives have commercial technical support. Similar to a previous study on healthcare professionals’ experiences within the study [30], participants highlighted basic device functionality troubleshooting, rather than AID specifics, as the biggest source of troubleshooting. Participants’ expert critiques identified structures within the New Zealand health system precluding equal access to diabetes technologies outside of the trial; namely insulin pump access criteria and healthcare professionals influencing technology advocacy and support. The later may explicate disparities in technology utilisation by geographic location. Compounding socioeconomic barriers to CGM access appeared to provide an even longer path of resistance to health equity for Māori.

Participants flourished when afforded the tools to manage T1D, and in recognition of this we propose strength-based solutions to structural factors restricting access to diabetes technologies for Māori, including funding and quality of clinical care. Insulin pumps, CGM, and AID systems should be publicly funded for Māori with T1D – effectively, ameliorating biases forced on healthcare professionals. Importantly, New Zealand should learn from the publicly funded insulin pump example, which illustrates that access criteria can amplify inequities [11, 14]. Consistent with other studies evaluating the experiences of Māori in the New Zealand health system [31,32], this study acknowledges the influence clinical teams have on health equity. Accordingly, diabetes clinical teams should be adequately resourced (in knowledge, cultural competency, and time) to support Māori to adopt and maintain emerging technologies. These recommendations align with other research addressing health equity for Māori with type 2 diabetes (T2D); Mana Tū, a whānau ora (family health) approach to T2D, similarly addresses individual, whānau, service, and system factors restricting health equity [33].

Existing literature has identified profound disparities in diabetes health outcomes, including access to diabetes technologies, for marginalised ethnic groups [34]. Further, research has determined that disparities in technology utilisation by ethnicity are not entirely encapsulated by socio-economic deprivation [11, 15]. However, to the best of our knowledge, this research is the first effort to understand the barriers to equity in diabetes technology utilisation in New Zealand. Other strengths of this study include the KMR design which privileges the expertise of Māori participants to identify solutions to barriers to health equity in T1D. Consequently, these findings are unique to Indigenous Māori and they may not be transferable to other contexts. Recruitment of Māori from the CREATE trial may have limited participation to those with greater access to the determinants of Indigenous health since they had to be using an insulin pump to access the CREATE trial. Similarly, their prior experiences with CGM may have influenced their response to adopting AID technology, compared to those who were novice CGM users. Findings may be limited further by lack of interview data from four of the 14 Māori participants in the CREATE trial who did not respond to invitations to be interviewed for reasons unknown to the researchers. Despite this, the sample of ten participants with additional insights from whānau provided the researchers with a great appreciation for the structures challenging equity for Māori with T1D.

In conclusion, use of an open-source AID system in the CREATE trial improved quality of life, wellbeing, and glycaemia for Māori with T1D. However, structural and socio-economic barriers preclude equitable utilisation of diabetes technologies for Māori outside of funded clinical trials. This research proposes solutions to the barriers to equity which should be considered in the strength-based redesign of diabetes health services to improve service provision for Māori with T1D.

## Supplementary Information

Below is the link to the electronic supplementary material.

Supplementary file1 (PDF 220 KB)

## Data Availability

Participant information and consent forms signed by all trial participants preclude the sharing of patient data beyond this trial.
