# Supplementary material for: Interviews with Indigenous Māori with type 1 diabetes using open-source automated insulin delivery in the CREATE randomised trial
Source: J Diabetes Metab Disord. 2023 Mar 23;22(1):861–71. doi: 10.1007/s40200-023-01215-3 (PMC10035484; doi:10.1007/s40200-023-01215-3)
Supplement: Supplementary file 1 — Supplementary file1 (PDF 220 KB) [file 40200_2023_1215_MOESM1_ESM.pdf]

Supplemental Appendix

Addressing the ethnic digital diabetes divide; interviews with Māori patients with type 1 diabetes using open-source automated insulin delivery in the CREATE randomised trial.

Submitted to the Journal of Diabetes and Metabolic Disorders

Mercedes Burnside,[1] Tracy Haitana,[2] Hamish Crocket,[3] Dana Lewis,[4] Renee Meier,[1] Olivia Sanders,[1] Craig Jefferies,[5,6] Ann Faherty,[5] Ryan Paul,[3,7] Claire Lever,[7] Sarah Price,[7] Carla Frewen,[8] Shirley Jones,[8] Tim Gunn,[9] Benjamin J Wheeler,[8] Suzanne Pitama,[2] Martin de Bock,[1] Cameron Lacey.[2]

1. Department of Paediatrics, University of Otago, Christchurch, New Zealand.
2. Department of Māori Indigenous Health Innovation (MIHI), University of Otago, Christchurch, New Zealand.
3. Te Huataki Waiora School of Health, University of Waikato, Hamilton, New Zealand.
4. OpenAPS, Seattle, WA, United States of America.
5. Department of Paediatric Endocrinology, Starship Children's Health, Te Whatu Ora Te Toka Tumai, Auckland, New Zealand.
6. Liggins Institute and department of paediatrics, University of Auckland, Auckland, New Zealand.
7. Waikato Regional Diabetes Service, Te Whatu Ora Health New Zealand Waikato, Hamilton, New Zealand.
8. Department of Women's and Children's Health, Dunedin School of Medicine, University of Otago, Dunedin, New Zealand.
9. Nightscout New Zealand, Hamilton, New Zealand.

Corresponding Author: Mercedes Burnside, Department of Paediatrics, University of Otago, Christchurch, 4 Oxford Terrace, Christchurch 8011, New Zealand; email address burme647@student.otago.ac.nz.

Contents

Supplemental Figure 1. 3
Supplemental Table 1. Application of Kaupapa Māori Research Principles. 4
References 6

Supplemental Figure 1.

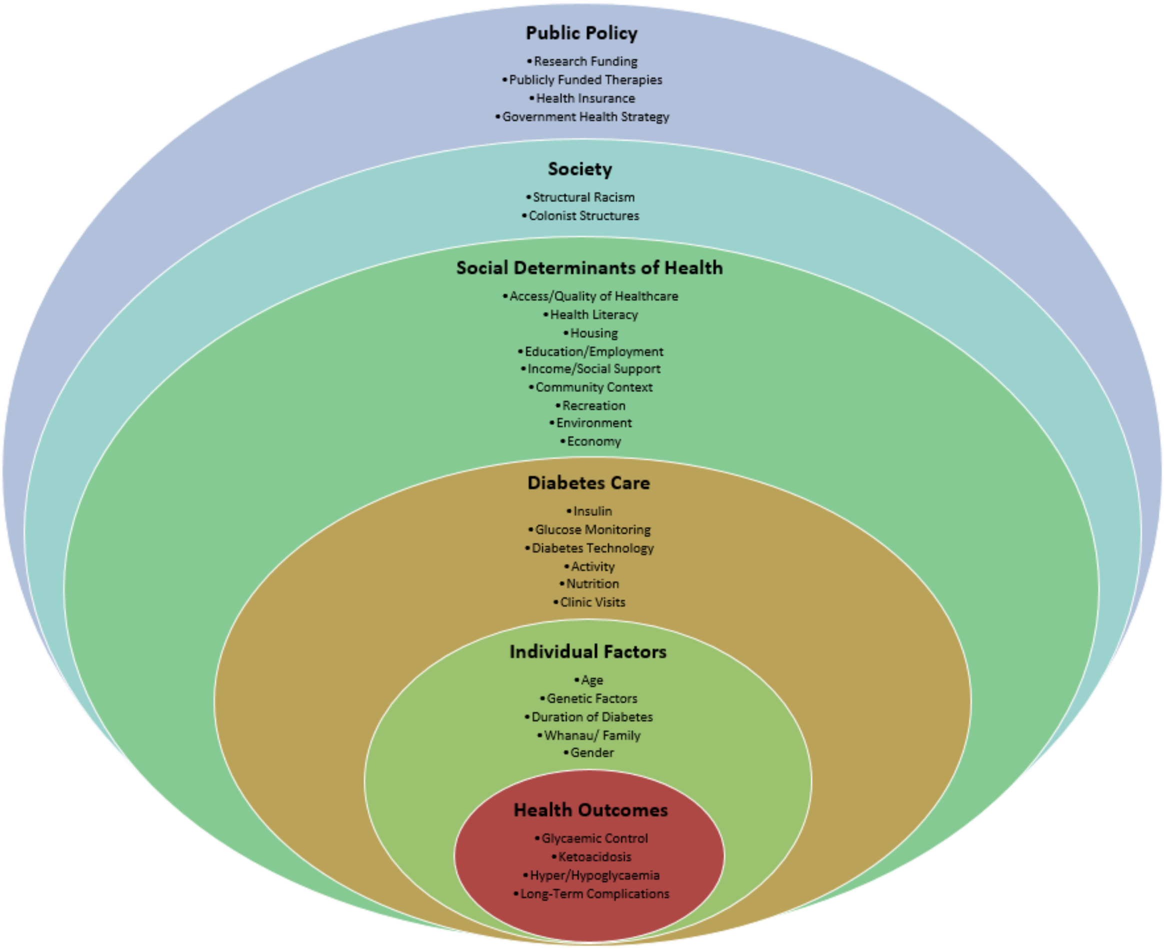

Supplemental Figure 1: Understanding Health Inequities (adapted from [1])

The present epidemiological profile of minoritized ethnic groups is associated with high deprivation indices, unemployment, lack of education, involvement in the criminal justice system, and marginalisation. Poor access to the social determinants of health understandably takes priority over intensive diabetes self-management and/or frequent diabetes clinic attendance. Interventions to lessen disparities in health outcomes must address also public policy, societal structures, and the social determinants of health.

Supplemental Table 1. Application of Kaupapa Māori Research Principles.

Principle	Description	Application
**Tino Ranagatiratanga**	Self-determination<br><br>Māori having meaningful control over their health/cultural wellbeing	Semi-structured interviews with open ended questions privileges the voices of Māori participants and positions the researchers as non-expert.<br>Participants have an intellectual position and feel empowered (whakamana) to share their lived experiences of the AID system; Māori become the theorizers.<br>The study represents a partnership between Māori researchers, Māori healthcare professionals and Māori people/whānau affected by T1D; there is Māori involvement in all levels of this research.
**Ako**	Culturally preferred pedagogy<br><br>Using teaching/ learning practices that are unique to Māori customs (tikanga)	Interviews followed a New Zealand Indigenous health framework [2].<br>Hui processes, specifically whakawhānaunagtanga (the process of establishing a meaningful relationship), created a safe physical, emotional, and spiritual space for discussion to occur. Face to face (kanohi ki te kanohi) interviews facilitated whakawhānaungatanga.
**Taonga Tuku Iho**	Cultural aspirations<br><br>Legitimate Māori language (Te reo), knowledge (matauranga), and customs (tikanga)	Researchers possessed knowledge of Māori epistemology – the Māori world view is holistic and health articulates physical, mental, spiritual and emotional elements from individual and collective perspectives.<br>Researchers validated Te reo through use.
**Kia Piki Ake i nga Raruraru i te Kainga**	Mediating socioeconomic factors<br><br>Addressing socioeconomic disadvantage	Efforts were made to minimise socioeconomic barriers to participating in interviews. Researchers travelled and interviews were undertaken at locations chosen by participants.<br>The CREATE trial allowed Māori to access diabetes technologies without financial cost.<br>Open-source AID systems aim to provide a cost-effective alternative to commercial systems.
**Whānau**	Extended family structure<br><br>The cultural values/customs that organise around the whānau and collective responsibility are important for Māori achievement.	Whānau were encouraged to be involved in interviews with participants, and their voices were regarded as equally important. This acknowledges that Māori with T1D do operate in a silo and establishing how the AID system impacted wider whānau was essential.<br>The presence of whānau may have further empowered participants to voice their views on use of the open-source AID system, and experiences with the health system more generally.
**Kaupapa**	A collective philosophy<br><br>Aspirations of the community	Given the burden of diabetes for Māori, improving diabetes care and health outcomes is a priority for Māori, and hence this research contributes to the overall Kaupapa.

**Supportive of Decolonisation**		A Māori philosophical and decolonising lens was applied to the analysis to avoid validating Māori as the problem or Māori being perceived as a risk factor or vulnerable.
**Transformative**		This research is not centered on improving glycaemic control for Māori. It hopes Māori expertise will co-determine systemic change that translates to equitable diabetes care and health outcomes for Māori – for example, increasing the interface with clinical care teams, greater access to technology, and mediating socioeconomic factors.
Kaupapa Māori Research (KMR) methodologies informed this qualitative work [3]<br>Māori – the Indigenous People of Aotearoa/New Zealand<br>Whānau – extended family<br>Hui Process – a framework to guide clinical interaction with Māori derived from a Māori world view<br>Kaupapa – collective vision/aspiration		

References

1. Lipman TH, Hawkes CP. Racial and Socioeconomic Disparities in Pediatric Type 1 Diabetes: Time for a Paradigm Shift in Approach. Diabetes Care. 2020;44(1):14-6.
2. Pitama S, Huria T, Lacey C. Improving Maori health through clinical assessment: Waikare o te Waka o Meihana. New Zealand medical journal. 2014;127(1393):107-19.
3. Pihama L, Cram F, Walker S. Creating methodological space: A literature review of Kaupapa Maori research. Canadian Journal of Native Education. 2002;26(1).
